# Genome Sequences of *Acholeplasma laidlawii* Strains Differing in Sensitivity to Ciprofloxacin

**DOI:** 10.1128/genomeA.01189-17

**Published:** 2017-11-02

**Authors:** Elena S. Medvedeva, Maria N. Siniagina, Sergey Y. Malanin, Eugenia A. Boulygina, Tatyana Y. Malygina, Natalia B. Baranova, Alexey A. Mouzykantov, Marina N. Davydova, Olga A. Chernova, Vladislav M. Chernov

**Affiliations:** a Kazan Institute of Biochemistry and Biophysics, Kazan Scientific Centre of the Russian Academy of Sciences, Kazan, Russia; b Institute of Fundamental Medicine and Biology, Kazan (Volga Region) Federal University, Kazan, Russia

## Abstract

*Acholeplasma laidlawii* is a well-suited model for study of the molecular basis of the adaptation of mollicutes to environmental conditions. Here we present the whole-genome sequences of four strains of *A. laidlawii* with differential sensitivity to ciprofloxacin.

## GENOME ANNOUNCEMENT

Bacteria of the class *Mollicutes* (the smallest prokaryotic organisms lacking a cell wall) are the commensals of higher eukaryotes; the parasites of plants, animals, and humans; and the main contaminants of cell cultures (1, 2). The recommended approach to suppress and eliminate mollicute infection and contamination is antibiotic therapy associated with the use of fluoroquinolones, including in combination with tetracycline and macrolides (3, 4). *Acholeplasma laidlawii* is a unique species among the *Mollicutes* in its adaptability. As *A. laidlawii* is widespread in nature (it can be found in humans, animals, plants, and cell cultures), and due to the relative simplicity of its cultivation as well as the availability of the complete genome sequence (*A. laidlawii* PG-8A) and the proteome under different growth conditions (5–8), the bacterium is a well-suited model for studies of the *Mollicutes*, including their development of antibiotic resistance *in vitro*.

Four strains of *A. laidlawii* with differing sensitivities to ciprofloxacin—a laboratory strain of PG8B (MIC 0.5 μg/ml) as well as strains PG8R_10_ (MIC 20 μg/ml), PG8R (MIC 1 μg/ml), and PG8S (MIC 0.2 μg/ml), which are derivatives of the PG8B strain—were sequenced in our study.

DNA from cells of the *A. laidlawii* strains was extracted using the phenol extraction method (9). Whole-genome sequencing of all strains was performed on the 454 Roche JS Junior platform. The libraries were prepared using a rapid library preparation kit and the GS Junior Titanium emPCR kit (Roche Diagnostics). The obtained reads, with 16.6-, 23.2-, 37.5- and 20.9-fold genome coverage, were assembled *de novo* using Newbler 2.6 (Roche Diagnostics), generating 21, 38, 15, and 19 scaffolds for PG8B, PG8R, PG8R_10_, and PG8S, respectively. Alignment to the reference genome of *A. laidlawii* PG-8A (GenBank accession number CP000896) was performed using the Bowtie 2 software (10). Gene predictions and annotations were performed using the NCBI Prokaryotic Genome Annotation Pipeline (11). The search and annotation of single nucleotide polymorphisms (SNPs) was performed using SAMtools (12) and SnpEff (13), respectively.

The *A. laidlawii* strains with increased resistance to ciprofloxacin showed single nucleotide polymorphisms (SNPs) in the genes of DNA gyrase and DNA topoisomerase. The identified point mutations are located in the quinolone resistance-determining regions (QRDR) of the *gyrA* and *parC* genes associated with the development of ciprofloxacin resistance in different microorganisms (14, 15). In addition, SNPs were identified in many other genes for which involvement in antibiotic resistance remains to be elucidated. Some SNPs found in PG8R and PG8R_10_ were also detected in the *A. laidlawii* strains with increased resistance to tetracycline (GenBank accession number NELO01000000) and melittin (GenBank accession number NELN01000000).

The whole-genome sequences of the *A. laidlawii* strains with differential sensitivity to ciprofloxacin can be used further to determine the molecular basis for adapting these bacteria to antibiotics.

### Accession number(s).

The whole-genome shotgun projects of PG8B, PG8R, PG8R_10_, and PG8S have been deposited in DDBJ/ENA/GenBank under the accession numbers LVCP00000000, LZGF00000000, LXYB00000000, and LZGE00000000, respectively. The versions described in this paper are the first versions, LVCP01000000, LZGF01000000, LXYB01000000, and LZGE01000000.
